# Vitamin D in tuberous sclerosis complex-associated tumors

**DOI:** 10.3389/fped.2024.1392380

**Published:** 2024-05-23

**Authors:** Tatsuro Nobutoki

**Affiliations:** Department of Pediatrics, Social Welfare Aiseikai, Suihoen, Japan

**Keywords:** tuberous sclerosis complex, tumor, 1,25-Dihydroxyvitamin D_3_, vitamin D receptor, mTOR

## Abstract

Mammalian target of rapamycin inhibitors (mTORi) have been used to treat pediatric tuberous sclerosis complex (TSC)-associated tumors, particularly in cases with contraindications to surgery or difficulties in complete tumor resection. However, some patients experience side effects and tumor regression after discontinuation of the treatment. Therefore, there is an urgent need to develop drugs that can be used in combination with mTORi to increase their efficacy and minimize their side effects. 1,25-Dihydroxyvitamin D_3_ (1,25-D), which has anticancer properties, may be a promising candidate for adjuvant or alternative therapy because TSC and cancer cells share common mechanisms, including angiogenesis, cell growth, and proliferation. Vitamin D receptor-mediated signaling can be epigenetically modified and plays an important role in susceptibility to 1,25-D. Therefore, vitamin D signaling may be a promising drug target, and *in vitro* studies are required to evaluate the efficacy of 1,25-D in TSC-associated tumors, brain development, and core symptoms of psychiatric disorders.

## 1 Introduction

Tuberous sclerosis complex (TSC) is an autosomal dominant disorder caused by inactivating mutations in either the *TSC1* or *TSC2* gene, which affects multiple organ systems and results in various clinical features (1). *TSC1* encodes *hamartin* and *TSC2* encodes *tuberin.* Since TSC1 and TSC2 are tumor suppressor genes, abnormalities in *hamartin* or tuberin lead to mammalian target of rapamycin (mTOR) overactivation and multisystemic cellular proliferation, migration, and differentiation abnormalities (1). The role of mTOR inhibitors (mTORi) in cancer (2) and their safety in TSC (3) have been established. However, for children with TSC-associated tumors, it is critical to have an alternative therapeutic option when mTORi are ineffective or cannot be used.

1,25-Dihydroxyvitamin D_3_ (1,25-D) activates DNA damage-inducible transcript 4 (DDIT4) (4), which activates the TSC1-TSC2 complex and ultimately represses mTOR (5). Daily vitamin D supplementation was shown to reduce overall cancer mortality (6). An association has been suggested between cancer and low levels of circulating 25-hydroxyvitamin D_3_ (25-D) in ovarian (7), prostate (8), and colorectal cancers (9). However, whether vitamin D can prevent cancer remains controversial. Therefore, it is important to investigate whether 1,25-D not only serves as an adjuvant therapy by inhibiting or reducing TSC-associated tumor cell proliferation but also improves mTORi tolerability.

In addition to its effects on mTOR suppression, 1,25-D negatively regulates energy metabolism in cancer cells, including glucose and lipid metabolism, protection from oxidative stress, and cancer progression (10). Higher serum 25-D levels are associated with a reduced risk of glioma in elderly men (11). Recent results from an *in vitro* study have led to a discussion on the potential clinical use of vitamin D for treating glioblastoma (12). Thus, 1,25-D may be a weak suppressor of TSC-associated tumor growth, including in cases of drug resistance and rapidly growing subependymal giant cell astrocytoma (SEGA). This article provides perspectives on the potential adjuvant therapy using 1,25-D in patients with TSC, along with a review and presentation of hypotheses associated with the underlying physiological mechanisms.

## 2 Need for an alternative or supportive drug to mTORi in pediatric TSC

In pediatric patients with TSC, SEGA (in ages ≥1 year) and refractory partial-onset seizures (as an adjunctive treatment, in ages ≥2 years) can be treated with mTORi (13). However, side effects are more common in children than in adults (13). The overall incidence of adverse events in children aged <9 years was 70.5% (24 of 34 patients), of which 33.3% (8 of 24 patients) had grade 3 side effects (13). Moreover, the mechanism of mTOR resistance in each tumor type has not been elucidated yet. Therefore, in addition to investigating the resistance mechanism, there is an urgent need to identify safe and effective drugs that can support mTORi treatment, including drug repurposing and combination therapy.

## 3 1,25-D inhibits vascular endothelial growth factor (VEGF) production and early angiogenesis in TSC-associated tumors

1,25 D causes transcriptional changes by binding to the intracellular vitamin D receptor (VDR). This binding forms a complex that interacts with specific DNA sequences called vitamin D-response elements (VDREs) located within the promoter regions of target genes (14).

**Figure 1:**
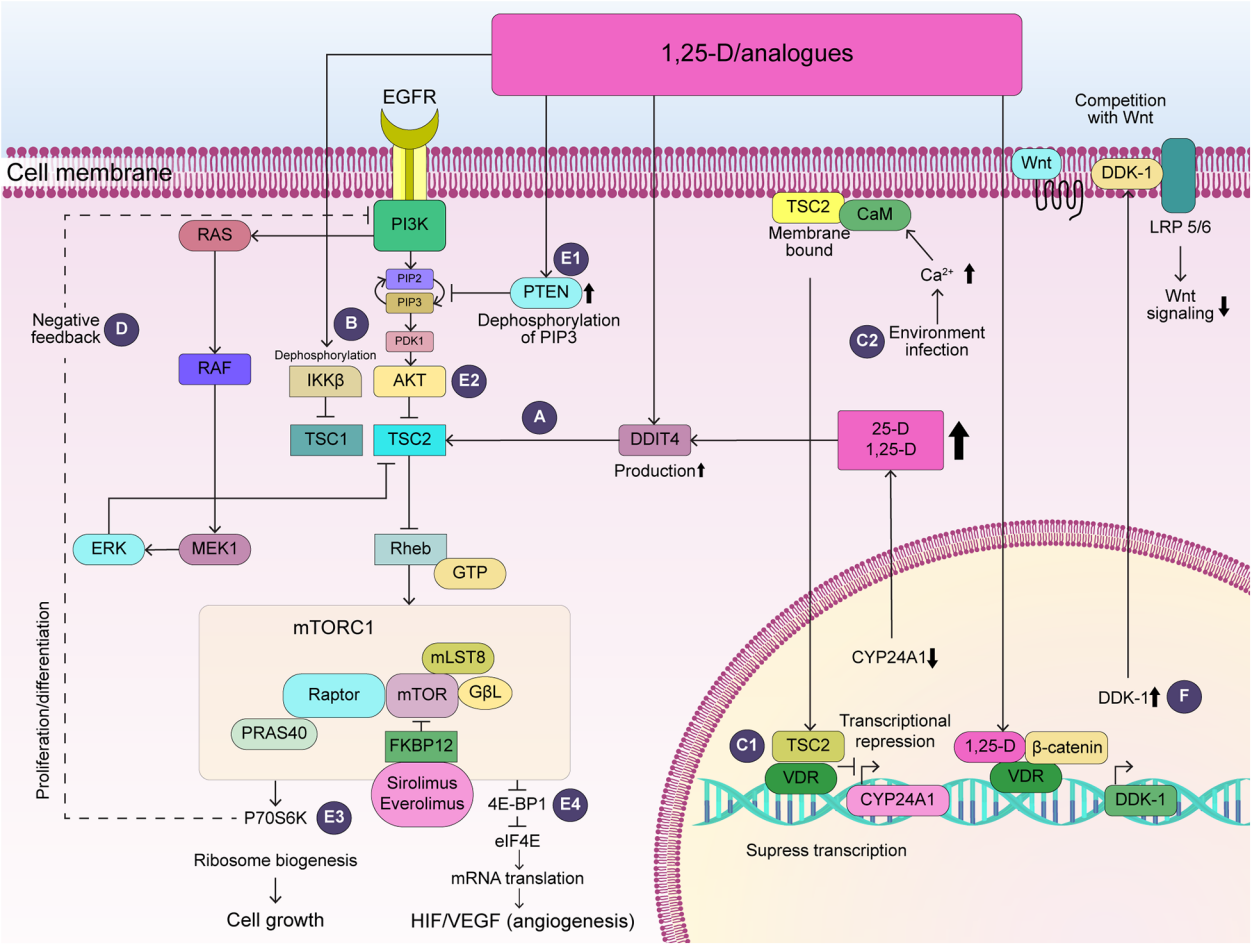
Potential action of vitamin D in TSC-associated tumors. (**A**) DDIT4 activates TSC2, which inhibits Rheb and ultimately suppresses mTOR activity. (**B**) 1,25-D dephosphorylates and inactivates IKKβ, which inhibits TSC1. (C1) TSC2-CaM complex translocates into the nucleus and represses CYP24A1 transcription. (C2) Increased intracellular calcium, which activates CaM by environmental factors including viral infection, could increase attenuation of transcription of VDR-sensitive genes. (**D**) S6K activation suppresses the PI3K/AKT and ERK pathway via negative feedback. (**E**) 1,25-D activates the production of PTEN (E1), which inhibits PI3K, leading to the dephosphorylation of Akt (E2), ultimately reducing p70S6K activity (E3) and 4E-BP1 (E4). Thus, 1,25-D may contribute to the suppression of cell growth and angiogenesis in TSC-associated tumors. (**F**) 1,25-D increases VDR/β-catenin binding, which increases transcription of the VDR-sensitive gene, DDK-1, competing with Wnt and decreasing Wnt signaling. AKT, RAC-alpha serine/threonine-protein kinase; CaM, calcium-calmodulin; CYP24A1, 24-hydroxylase; 1,25-D, 1,25-dihydroxyvitamin D_3_; DDK-1, Dickkopf-1; DDIT4, DNA damage inducible transcript 4; Deptor, DEP-domain-containing mTOR-interacting protein; 4E-BP1, eukaryotic initiation factor 4E-binding protein 1; eIF4E, eukaryotic initiation factor 4E; EGFR, epidermal growth factor receptor; ERK1-2, extracellular signal-related kinase; FKBP12, FK506-binding protein with a molecular weight of 12 kDa; GβL, G protein β subunit-like protein; GTP, guanosine triphosphate; HIF-1α, hypoxia inducible factor-1-alpha, IKKβ, IκB kinase β; Lamptor, late endosomal/lysosomal adaptor, mitogen-activated protein kinase, and mTOR activator; LRP5/6, low-density lipoprotein receptor-related proteins 5 and 6; MEK1-2, mitogen-activated protein kinase 1-2; mLST8, mammalian lethal with SEC13 protein 8; mTOR, mammalian target of rapamycin; mTORC1, mTOR complex 1; P70S6K, 70-kDa ribosome protein S6Kinase; PDK1, 3-phosphoinositide-dependent protein kinase-1; PIP2, phosphatidylinositol 4,5-bisphosphate; PIP3, phosphatidylinositol-3,4,5-trisphosphate; PI3K, phosphoinositide 3-kinase; PRAS40, proline-rich Akt substrate of 40 kDa; PTEN, phosphatase and tensin homologue; RAF, rapidly accelerated fibrosarcoma viral oncogene homologue; RTK, rector of tyrosine kinase; Raptor, regulatory associated protein of mTOR complex 1; RAS, rat sarcoma viral oncogene homologue; Rheb, Ras homologue enriched in the brain; TSC, tuberous sclerosis complex; VDR, vitamin D receptor; VEGF, vascular endothelial growth factor.

1,25-D activates the production of the DDIT4 protein, which is induced by hypoxia and DNA damage via intracellular VDR (5). The DDIT4 inhibits mTOR complex 1 by promoting TSC1-TSC2 complex formation (15) (Figure 1A). Moreover, the DDIT4-TSC1/TSC2-mTOR feedback loop downregulates the production of hypoxia-inducible factor-1α (HIF-1α) and VEGF (Vascular Endothelial Growth Factor) (15). siRNA knockdown of DDIT4 eliminates the antiproliferative effect of 1,25-D (16). Thus, 1,25-D restricts HIF-1-dependent VEGF production in various human cancer cells under hypoxic conditions (17) and induces apoptosis in existing sprouted and elongated endothelial cells (18) Notably, TSC-associated tumors are angiogenic neoplasms (19) expressing high levels of VEGF (20). Through this mechanism, 1,25-D may suppress tumor cell proliferation by inhibiting the excessive activation of the HIF/VEGF pathway in the vasculature of TSC-associated tumors.

Moreover, activation of VEGF, HIF, and endothelial cell-dependent mechanisms contribute to vasculogenic mimicry (VM) in cancer (21). This involves the formation of microvascular channels composed of tumor cells that contribute to the resistance to anti-angiogenic therapy (22). Hypoxic conditions and the aberrant activation of the mTOR/HIF/VEGF pathway can occur in cancer and TSC-associated tumors. Indeed, astrocytomas have a VM-like structure (23), and VEGF- and HIF-dependent factors play an important role in their pathogenesis (20). This mechanism may contribute to therapy resistance in SEGA and to tumor regrowth after discontinuation. Therefore, it is reasonable to target the HIF/VEGF pathway with 1,25-D since a VM-like structure may be formed in TSC-associated tumors, including SEGA.

TSC1 is suppressed by IκB kinase β (IKKβ) phosphorylation, leading to the activation of the mTOR pathway and increased VEGF production and angiogenesis (24). 1,25-D induces direct VDR-IKKβ protein interaction, disrupting the formation of the IKK complex, which consists of IKKα, β, and *γ* subunits, and abolishes IKKβ phosphorylation (25) (Figure 1B). Through this mechanism, 1,25-D suppresses VEGF-mediated angiogenesis.

## 4 Impaired interaction between TSC2 and VDR may contribute to TSC severity

In tumor-derived endothelial cells, the transcription of one of the VDR target genes, 24-hydroxylase (CYP24A1) is upregulated and this promotes the metabolism of 25-D and 1,25-D. The activation of CYP24A1 promotes 25-D metabolism, thereby potentially reducing the cellular availability of 1,25-D. Moreover, cell cycle arrest, growth inhibition, and apoptosis are induced by epigenetic silencing of CYP24A1 (26). Importantly, plasma membrane-bound TSC2 binds to calcium/calmodulin (CaM), and this complex is translocated to the nucleus, partially attenuating CYP24A1 transcription under normal conditions (27) (Figure 1C1). Mutations in TSC2 lead to more severe clinical features compared to those in TSC1 This, in turn, could contribute to the severity of TSC and the formation of TSC-associated tumors. This, in turn, could contribute to the severity of TSC and the formation of TSC-associated tumors. Therefore, along with inhibiting angiogenesis and regressing existing immature capillaries, 1,25-D treatment produces effects in tumor cells similar to those of epigenetic silencing of CYP24A1.

## 5 Second hit by epigenetic alterations in the vitamin D metabolism and VDR signaling pathway may be associated with clinical features in TSC

The correction of histone hyperacetylation in hippocampal neurons using histone deacetylase (HDAC) inhibitors improves abnormal synaptic plasticity and epilepsy in TSC (28). While second-hit mutagenesis may play an important role in the phenotypic diversity of renal lesions in TSC (29), the VDR and VDR-responsive genes can be epigenetically modified (30), similar to histone hyperacetylation in neurons. Therefore, postnatal epigenetic modifications of VDR-mediated signaling and vitamin D metabolism may serve as a second hit and contribute to the clinical variability and severity of TSC, including the development of tumors, abnormal synaptic plasticity, and epilepsy. As discussed above, CaM plays an important role in the TSC2-VDR interaction. Therefore, increased intracellular calcium and CaM activation caused by viral infection could increase the attenuation of VDR-sensitive gene transcription, including that of CYP24A1, by enhancing the TSC2-CaM interaction or inducing epigenetic modifications that affect the transcriptional activity of VDR-related genes (Figure 1C2).

Epigenetic silencing of VDR, which is also mediated by HDAC3, is crucial in 1,25-D resistance (30), and the treatment of VDR promoter hypermethylation with 5-aza-2′-deoxycytidine restored VDR mRNA expression (31). In addition, hypermethylation of CYP27B1, which activates 25-D to 1,25-D, can reduce the tissue levels of 1,25-D (30). High blood 25-D and 1,25-D levels may be required in this condition.

## 6 SEGA responds to 1,25-D under increased VDR expression and sensitivity

Although VDR expression is increased in human glioblastoma (GB) cells (32), high VDR expression in GB is associated with 1,25-D treatment success (33). In an *in vitro* study, calcitriol and vitamin D analogue blocked the stem-like properties of glioma cells (34), induced DNA fragmentation (35), and inhibited the migration of GB cells (33). Furthermore, 1,25-D restores responsiveness to itself by upregulating VDR expression (36). Based on these observations, VDR expression in SEGA potentially correlates with 1,25-D sensitivity or aggressiveness. It's also speculated that SEGA, exhibiting resistance or recurrence to mTOR inhibitors, may respond to 1,25-D similar to GB.

Vitamin D_3_ analogue can suppress the activation of the phosphatidylinositol 3-kinase (PI3K)/RAC-alpha serine/threonine-protein kinase (AKT) pathway and extracellular signal-regulated kinase (ERK)/Mitogen-activated protein kinase (MAPK) pathway due to mTORi therapy.

S6Kinase (S6K) activation via mTORC1 suppresses the PI3K/AKT and ERK pathways via negative feedback (Figure 1D). Therefore, S6K inhibition by mTORi contributes to the activation of both pathways, thereby contributing to the pathology of mTORi-resistance. Therefore, combination therapy with MAPK inhibitors may be useful (37). Notably, vitamin D and its analogs restrict gliomas by inducing cell cycle arrest via multiple mechanisms, including the PI3K/AKT pathway (12). Importantly, while therapy with mTORi and PI3Kα inhibitor is necessary for GB treatment (38), activation of the PI3K/AKT (39) and MAPK/ERK pathways (40) also plays an important role in SEGA development. Moreover, in TSC, the Raf-1/MAPK/ERK cascade, in addition to mTOR, leads to 4E-binding protein 1 phosphorylation, increased cyclin D protein levels, and increased protein synthesis (41). The active vitamin D analog, maxacalcitol, decreased hyperphosphorylation of MAPK-38p and ERK1/2 in the brain tissue of a mouse model of Alzheimer's disease (42). Therefore, the vitamin D_3_ analog may suppress the growth of renal cancer and SEGA in the same way. Particularly in patients with mutations in the tumor suppressor gene phosphatase and tensin homologue (PTEN), PI3K/AKT activation leads to tuberin phosphorylation and decreased activity of tuberin-hamartin complex, resulting in the activation of mTOR/70kDa-S6K1 signaling (43). A novel vitamin D_3_ analog, Gemini-23-yne-26,27-hexafluoro-D_3_, not only increased the expression of PTEN and caused the dephosphorylation of Akt, Ark target proteins, and mTOR, but also decreased the phosphorylation of its downstream effectors, S6Ks, and eukaryotic translation initiation factor 4E-binding protein 1, thereby suppressing protein synthesis and tumor proliferation (44) (Figures 1E1–E4). Similarly, tumors associated with Tuberous Sclerosis Complex (TSC), such as SEGA, might see improved treatment outcomes with novel vitamin D derivatives. These derivatives activate PTEN, suppress the PI3K/AKT/mTOR pathway, and enhance the effectiveness of mTOR inhibitors, potentially leading to safer and more effective therapies compared to using mTOR inhibitors alone.

## 7 Suppressing wingless/int-1 (Wnt)/β-catenin signaling

Abnormal Wnt/β-catenin signaling plays an important role in tumorigenesis (45). The TSC1-TSC2 complex negatively regulates cell proliferation through β-catenin signaling (46), which plays an important role in the pathogenesis of angiomyolipomas and lymphangioleiomyomatosis (47). Importantly, 1,25-D increases VDR/β-catenin binding, which, in turn, increases the transcription of one of the VDR target genes, the Wnt inhibitor Dickkopf-1, to a greater degree than that of the T-cell factor (48) (Figure 1F). As a result, 1,25-D decreases the transcription of β-catenin/T-cell factor-target genes that regulate cell proliferation, cell cycle regulation, and cellular metabolism (48). Therefore, 1,25-D may inhibit the growth of TSC-associated tumors.

## 8 Identification of 1,25-D-induced microRNAs (miRNAs) that suppress the growth of TSC-associated tumors and re-sensitize mTORi-resistant tumors

miRNAs are short noncoding RNAs with a wide range of gene regulatory activities at the post-transcriptional level (49). 1,25-D re-sensitizes everolimus-resistant hepatocellular carcinoma by upregulating miRNA-375, which regulates the oncogenes responsible for drug resistance (50). In addition, miRNA-22 mediates the suppression of several genes by 1,25-D, contributing to its antiproliferative and antimigratory effects in colon cancer cells (49). Therefore, it's worth exploring if 1,25-D also triggers miRNA-mediated antitumor effects in TSC and enhances the effectiveness or sensitivity to mTORi.

## 9 Initiating 1,25-D therapy in infancy improves brain development in TSC

Vitamin D is essential for brain development (51), and loss of TCS2 function in brain endothelial cells, neurons, oligodendrocytes, astrocytes, and microglia may increase the degradation of vitamin D and decrease its bioavailability by the same mechanism discussed above. Therefore, central nervous system vitamin D deficiency in TSC might be one of the drivers of impaired neural and synaptic maturation, as well as of tumorigenesis. Notably, blood levels of 25-D higher than 40 ng/ml can improve the autism spectrum disorder (ASD) rating (52). Thus, the same therapeutic effects can be expected in TSC. If the sensitivity of neural and brain endothelial cells to vitamin D is increased, early vitamin D treatment from infancy can improve brain development and pediatric TSC-associated neuropsychiatric disorders, including the core symptoms of ASD and refractory epilepsy.mTOR-dependent synaptic hyperconnectivity is implicated in ASD pathogenesis in *Tsc2^+/^*^−^ mice (53). mTORi are effective in rescuing synaptic hyperconnectivity and controlling autistic behavior (53) and epilepsy in patients with TSC (54).

Neurological problems can be associated with decreased 1,25-D bioavailability in neurons and neuronal unresponsiveness to 1,25-D in the developing brain, owing to the epigenetic silencing of VDR-mediated signaling. In either case, the combination of mTORi and 1,25-D may synergistically improve brain development.

In addition, 1,25-D inhibits IKKβ phosphorylation and suppresses nuclear factor-κB (25), which plays an important role in the switch from oxidative stress to inflammation that contributes to epileptogenesis (55). Thus, 1,25-D may be promising for the reversal of TSC-associated brain pathological conditions and may play a role in suppressing the development of SEGA and reducing the clinical severity of comorbid neurological disorders.

## 10 Potential considerations in vitamin D treatment: therapy resistance and tumor growth

Since mTOR inhibition provides survival advantage for tumor cells (56), it is important to consider the risk of 1,25-D increasing tumor growth by inhibiting mTOR through DDIT4 activation. In silico analysis has shown that high DDIT4 expression, but not PI3K/mTOR activation, is associated with poor prognosis in some cancers, suggesting that DDIT4 inhibitors may be effective in these cases (56).

Tumor hypoxia, a condition in which solid tumors have hypoxic regions with an insufficient oxygen supply, contributes to resistance to chemotherapy (57). This may also occur in TSC-associated tumors. Under hypoxia, HIF-1α production is activated, which transcriptionally upregulates DDIT4 through a negative feedback loop of suppression against mTOR inhibition (15). Thus, when DDIT4 is overexpressed in TSC-associated tumors, mTOR/HIF-1α may persistently be overactivated by this mechanism, leading to a resistance to mTORi and 1,25-D. Under these conditions, DDIT4 inhibitors, but not 1,25-D, can minimize the dose of mTORi and avoid the need to discontinue treatment owing to side effects. Therefore, *in vitro* studies are important to determine the effectiveness of 1,25-D or its potential to exacerbate tumor progression, particularly in cases of high DDIT4 levels within the tumor tissue.

Moreover, while cholecalciferol exerts beneficial antidepressant effects through the activation of brain-derived growth factor/tyrosine receptor kinase B (TrkB) signaling in the prefrontal cortex (58), TrkB signaling plays an important role in TSC-associated neuropsychiatric disorders and epileptogenesis (59). While activation of the brain-derived neurotrophic factor/TrkB pathway by early 1,25-D therapy may improve brain development in children with TSC, epidemiological studies are required to investigate whether long-term 1,25-D treatment may improve neuropsychiatric disorders and epilepsy.

## 11 Epidemiological studies using large medical datasets may be useful for testing hypothesis

Considering the above discussion, it cannot be excluded that the clinical phenotypic variability in TSC, including tumor development, progression, and neurological problems, is partly due to the second hit in epigenetic modification of VDR and vit D-metabolizing enzyme genes by the environment, as well as dietary and supplemental vitamin D intake, use of multiple anticonvulsants, and reduced sun exposure. It is necessary to investigate whether 1,25-D treatment can reduce the risk or worsen the severity of TSC-associated tumors, refractory epilepsy, and neuropsychiatric conditions, including the core symptoms of ASD.

Analyzing medical big data or conducting retrospective studies on tumors, cancer incidence, prognosis, cognitive performance, and epilepsy severity in patients who received 1,25-D therapy compared to those who didn't, including assessing blood 25-D levels and the duration of 1,25-D therapy, may help determine if early and long-term treatment can benefit patients with TSC and TSC-associated tumors. Additionally, comparing the efficacy and side effects of mTORi alone vs. in combination with 1,25-D could provide valuable insights. However, conducting prospective studies raises ethical concerns due to the essential role of vitamin D in bone growth and immunity. Therefore, the analysis of medical big data could contribute to the discussion of the advantages and disadvantages of early 1,25-D therapy in patients with TSC.

## 12 Discussion

Since vitamin D has beneficial effects on several signaling pathways involved in the mechanism of TSC-associated tumors, 1,25-D and its analogs may be the first treatment choice. In children with TSC who have undertaken polytherapy of antiepileptic drugs, if vitamin D supplementation can not only prevent or slow tumor development but also improve brain development and reduce the core symptoms of TSC-associated neuropsychiatric disorders, it should be started immediately after diagnosis, preferably in infancy, in and added to anticonvulsant therapy. In addition, the possible increased metabolism of 25-D in TSC-associated tumors and the downregulation of the transcriptional activity of VDR-sensitive genes at a relatively high dose (blood levels of 25-D > 40 ng/ml) seems reasonable. As 1,25-D is relatively inexpensive and safe, children who are intolerant to mTORi, those in long-term care facilities, those receiving home care, or those unable to receive expensive medical care may benefit from 1,25-D therapy. Thus, 1,25-D may be a promising drug candidate for enhancing the effects of mTORi and improving tolerability, although it is also important to study whether it has any side effects. Particularly, the study of VDR signaling, including the epigenome, in TSC may have implications for drug discovery. Therefore, it is necessary to study vitamin D signaling and search for novel vitamin D analogs that are more effective and have fewer side effects, such as hypercalcemia in TSC-associated tumors.

*In vitro* studies are required to evaluate the efficacy of 1,25-D in TSC associated tumors, brain development, and core symptoms of psychiatric disorders.

## Data Availability

The original contributions presented in the study are included in the article/Supplementary Material, further inquiries can be directed to the corresponding author.
